# Graves’ disease post-COVID-19 m-RNA vaccine in pediatric age group 

**DOI:** 10.22038/AOJNMB.2023.73051.1510

**Published:** 2024

**Authors:** Abhaa Sulaiyam Al-Jahhafi, Asma Ali Al-Sawaai, Zamzam Khalifa Al-Bimani, Naima Khamis Al-Bulushi

**Affiliations:** 1 Oman Medical Specialty Board (OMSB), radiology residency Program, Oman; 2 Nuclear Medicine Department and Molecular Imaging Centre, Royal Hospital, Oman

**Keywords:** Thyroiditis, COVID-19 Vaccine, Graves' disease

## Abstract

The surge of the COVID-19 pandemic (December / 2019 - May/2023) and its catastrophic effect worldwide have necessitated emergent intervention to reduce its influence on people's health and life. To eliminate and reduce the impact of COVID-19 infection, COVID-19 vaccination was emergently authorized in December 2020 which has established good safety and efficacy. Having said that, some adverse effects merged in a few individuals. We are reporting an adolescent patient a 17-year-old female who has been diagnosed with Graves’ disease after post-COVID-19 vaccinations. In addition, she was a confirmed case of COVID-19 infection three months earlier. The patient presented with typical features of hyperthyroidism 30 days post receiving the first dose of the vaccination. Based on the patient's presentation relative to the administration of the vaccine and prior infection of the virus. We proposed the synergistic effect of both factors to induce Graves’ disease in this young healthy female with no family history of autoimmune disease. We are reporting this case for pediatric endocrinologists to be aware of the interaction and possible impact of the COVID-19 vaccine on thyroid function.

## Introduction

 Graves' disease (GD) is uncommon in childhood and adolescence when compared to adults, affecting 0.1 per 100,000 children and 3.0 per 100,000 adolescents per year. However, it is still considered the most common cause of hyperthyroidism in this age group (1). Although coronavirus disease (COVID-19) is primarily a respiratory disease, thyroid dysfunction, and autoimmune thyroid disorders were observed after the SARS-CoV-2 infection as well as after the introduction of COVID-19 vaccination. The COVID-19 pandemic has been associated with a higher occurrence of Graves’ disease relative to time before the pandemic as well there was an increase in the severity of symptoms of Graves’ disease after the pandemic (2). 

 Vaccination against COVID-19 proved to be safe and effective, with rare serious effects. It provides 70-95% protection against severe infection (3-5). Like other vaccinations, the participant reported numerous adverse symptoms following the COVID-19 vaccination, including muscle pain, fever, headache, nausea, and vomiting. Recently there are reported cases of thyroid dysfunction and autoimmune disease after the first or second doses of COVID-19 vaccination; however, the exact mechanisms were not yet fully clarified. Information is rapidly evolving about the impact of SARS-CoV-2 vaccines on the immune and endocrine systems; however, the vaccine has not been considered as a precipitating factor of thyroid dysfunction. We are reporting this case to enhance awareness of the possible impact of the COVID-19 vaccine on thyroid function in the pediatric group.

## Case presentation

**Figure 1:**
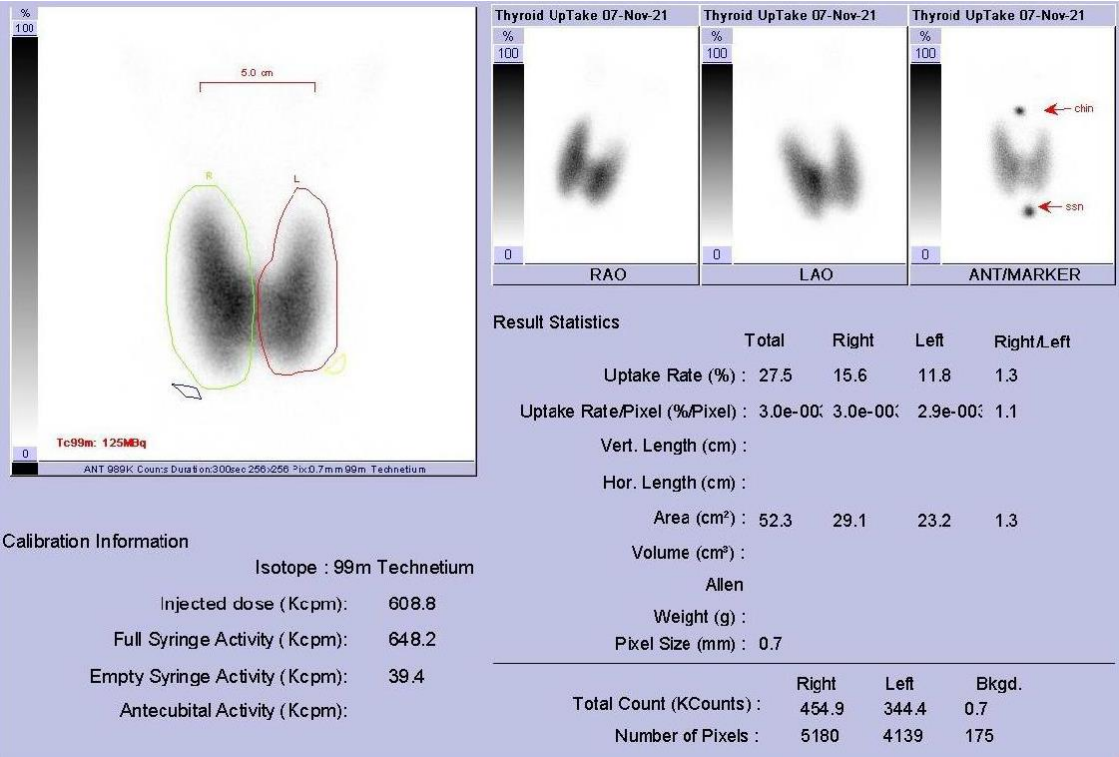
Tc-99m pertechnetate thyroid scintigraphy (thyroid scan), reveals diffusely increased radiotracer uptake in bilateral thyroid lobes, with suppression of the background activity. Global thyroid uptake was 27.5 %

 A 17-year-old female with no significant medical background presented with palpitation, hand tremors, and excessive sweating for a few weeks. She had mild shortness of breath associated with neck swelling. She had a PCR-confirmed COVID-19 infection three months prior to the onset of these symptoms that was mild and did not require hospital admission. In addition, the patient received the COVID-19 vaccine (Pfizer-BioNTech) one month prior to her presentation. On examination, she was found to have tachycardia 146 beat/minute), normal blood pressure (120/70 mmHg), and maintained saturation on room air. She had a fine tremor on both hands. Minor exophthalmos was noted on eye examination. However, the patient had no eye symptoms such as double vision. She had anterior neck swelling that was moving when swallowing, firm, and not tender. Her lab investigation as follows: HGB=9.36 g/dL (normal range: 11.5-16.5), White blood cells (WBC)=11.4×10^3^/uL ( normal 4 – 11), Platelets counts =227.8×10^3^/uL ( normal 150-450), Free (T4 )>100.00 pmol/L (normal range: 8.4-22.6), TSH <0.01 m[iU]/L (normal range: 0.27-5) Free (T3) =>50.00 pmol/L (normal range: 3-5.9), thyroid Peroxidase Antibody >1300.0 [iU]/ml. Thyroid ultrasound revealed bilaterally enlarged thyroid lobes with heterogeneous parenchyma and increased vascularity. No abnormal enlarged lymph nodes were noted. The patient was referred for thyroid scintigraphy which revealed diffusely increased radiotracer uptake in bilateral thyroid lobes, with suppression of the background activity. Global thyroid uptake (using Tc-99m pertechnetate) was 27.5 % (Normal 0.4-4%) (Figure 1). No TSH receptor antibodies (TRAB) levels are presented in this patient making the diagnosis of Graves' disease merely based on clinical findings and imaging findings of thyroid scintigraphy. The patient was started on Graves’ disease medication; Carbimazole 5 Mg three times a day and Propranolol 40 Mg three times a day and her symptoms resolved. One year follow-up of the patient revealed she is doing well, symptom-free with no progression in the minor bilateral exophthalmos but still on the same antithyroid medications.

## Discussion

 In December 2020 the emergency use authorization allows the Pfizer-BioNTech COVID-19 Vaccine to be distributed in the United States of America for individuals 16 years or older as the first vaccine for the prevention of coronavirus disease 2019 (COVID-19) caused by severe acute respiratory syndrome coronavirus 2 (SARS-CoV-2). COVID-19 vaccines had good effectiveness against COVID-19-related hospitalization, admission to the intensive care unit, and death in a real-world setting (6). As well it has been found that the COVID-19 vaccine reduces the risk of long COVID-19 sequelae. The protective effect was found in fully vaccinated individuals who received two doses regardless of whether before or after COVID-19 infection (7). The long COVID affects multiple organs, and common symptoms include tiredness/fatigue, dyspnea/difficulty breathing, cough, chest pain, diarrhea, headache, impaired balance and gait, insomnia, joint pain, myalgia, and weakness, neurocognitive issues, palpitations, pins and needles, rash, and hair loss (7).

 After the introduction of COVID-19 vaccination strategies, there have been reported cases in adult literature that propose an increase in Graves’ disease after vaccination. In the pediatric age group, we found that there have been only two published case reports of adolescents who developed Graves’ disease after receiving two doses of (Pfizer-BioNTech) vaccines. Their age was 15 and 14 years old, and both receive two doses of vaccine. Their symptoms developed 2 to 30 days after the second dose and were reported as atypical symptoms of Graves’ disease. One of the patients presented with lower extremity weakness and was found to have hypokalemia. Both patients had thyroid hyperactivity and positive thyroid-stimulating immunoglobulin (TSI), which were found incidentally (8). In adults, the median post-vaccination symptom onset was reported to be 10 days after the administration of the vaccine irrespective of the dose (9). Moreover, a recently published article described a case of a male patient who developed Graves’ disease within weeks of COVID-19 infection and 22 days prior he received the first dose of the Pfizer-BioNTech COVID-19 vaccine (10). In our reported case the patient developed typical features of hyperthyroidism 30 days post (Pfizer-BioNTech) vaccine, and 90 days earlier was a confirmed case of COVID-19 infection. So, it was difficult to determine whether the trigger for Graves' disease in this pediatric patient was the COVID-19 infection or an adverse event of the first dose of the BNT162B2 SARS-CoV-2 (Pfizer-BioNTech) vaccine, or whether it was a coincidence. Up to the present moment, 57 cases of Graves’ disease following COVID-19 vaccination were identified; two cases were previously infected with SARS-CoV-2. Most reported cases received BioNTech, Pfizer vaccine 64% (11). According to Herczeg et al, autoimmune thyroiditis was slightly more common in patients who had been infected with SARS-Cov-2 before, compared to the general pediatric population (12).

 It has been well known that viral infections are considered environmental factors that play a central role in the pathogenesis of autoimmune thyroid disorders (13). Many COVID-19-induced autoimmune diseases including autoimmune hemolytic anemia, Guillain–Barré syndrome, and autoimmune thrombocytopenic purpura have been reported since the SARS-CoV-2 outbreak in December 2019 (13). It is believed that the SARS-CoV-2 infection triggers a hyper-inflammatory cascade, or a “cytokine storm”-mediated autoimmune effects, that has also been found to be regulated particularly by type1 T helper cells (Th1) and IL-6 (14). Other factors that have been recently implicated to induce Graves' disease are called immune-related adverse events (irAEs) (15).

 On the other hand, the exact mechanism behind the potential association between COVID-19 vaccination and Graves’ disease still needs more clarification, several theories have been suggested. Autoimmune/inflammatory syndrome induced by adjuvants (ASIA) is the most frequently cited theory (16). This syndrome was first described in 2011 as an entity that incorporates diverse autoimmune conditions triggered by various adjuvants. Adjuvants are used to increase the immune response to the active substance and although essential for adequate immune system stimulation, they have been considered the etiological factor of ASIA following Hepatitis B and HPV immunization in the past most likely due to an intense immune response or genetic predisposition (17). Although mRNA vaccines do not use adjuvants, they contain lipid nanoparticles that facilitate mRNA transport into cells and could potentially induce immune responses in predisposed people. As this patient was also infected with COVID-19 90 days prior to the onset of symptoms, oxidative stress plays a role in the pathogenesis of Graves' disease (18).

 It is also worth mentioning that there are other thyroid disorders in the pediatric age group that were also reported in the recent literature and linked to the BNT162B2 SARS-CoV-2 (Pfizer-BioNTech) vaccine, namely thyroiditis (8). Hence, it is advised for pediatricians to be careful and watchful for potential post-vaccination thyroid disorders.

***Limitations***

### Limitations

 There are a few limitations in this case report that the authors would like to highlight. It is well known that Graves' disease has an accumulation of serum autoantibodies, the most important of which is the TSH receptor antibody (19). However, serum TSH receptor antibody was not available in this case making the diagnosis of Graves' disease merely based on clinical and imaging findings. Also, this case was referred to our center for imaging and is being managed and clinically followed in another health institution. Hence, a detailed and exact clinical response post-therapy was not possible as the patient was seen after one year of diagnosis in the referring hospital, and followed up of her medications was done in a primary health center.

## Conclusion

 Although the WHO has recently downgraded the Covid-19 pandemic (December 2019 – May 2023) from the status of a global emergency. The sequelae of this pandemic and the impact of the use of the vaccine on adults in general and on the pediatric age group, in particular, are yet to be revealed.

## References

[1] Lee HS, Hwang JS (2014). The treatment of Graves’ disease in children and adolescents. Annals of Pediatric Endocrinology Metabolism..

[2] Donner JR, Has P, Topor LS (2023). Increased incidence and severity of new Graves’ disease diagnoses in youth during the COVID-19 pandemic. Endocr Pract..

[3] Livingston EH, Malani PN, Creech CB (2021). The Johnson &amp; Johnson vaccine for covid-19. JAMA..

[4] Mulligan MJ, Lyke KE, Kitchin N, Absalon J, Gurtman A, Lockhart S (.2020). Phase I/II study of COVID-19 RNA vaccine BNT162b1 in adults. Nature.

[5] Barrett JR, Belij-Rammerstorfer S, Dold C, Ewer KJ, Folegatti PM, Gilbride C (2021). Phase 1/2 trial of SARS-CoV-2 vaccine ChAdOx1 nCoV-19 with a booster dose induces multifunctional antibody responses. Nat Med ..

[6] Zheng C, Shao W, Chen X, Zhang B, Wang G, Zhang W (2021). Real-world effectiveness of COVID-19 vaccines: A literature review and meta-analysis. Int. J. Infect. Dis..

[7] Gao P, Liu J, Liu M (2022). Effect of covid-19 vaccines on reducing the risk of long COVID in the real world: A systematic review and meta-analysis. International Journal of Environmental Research and Public Health..

[8] Chimatapu SN, Ferber CJ, Thambundit A, Okawa ER (2023). Two cases of thyroiditis in adolescents following COVID-19 vaccinations. JCEM Case Reports..

[9] Triantafyllidis KK, Giannos P, Stathi D, Kechagias KS (2022). Graves‘disease following vaccination against SARS-CoV-2: A systematic review of the reported cases. Frontiers in Endocrinology..

[10] Hamouche W, El Soufi Y, Alzaraq S, Okafor BV, Zhang F, Paras C (2022). A case report of new onset Graves’ disease induced by SARS-CoV-2 infection or vaccine?. Journal of Clinical and Translational Endocrinology: Case Reports..

[11] Shi S, Liang Z, Sun B (2020). Response to comment on: Vaccine adjuvants: Understanding the structure and mechanism of adjuvant city. Vaccine..

[12] Herczeg V, Garai R, Takács J (2023). Thyroid disturbances after COVID-19 and the effect of vaccination in children: a prospective tri-center registry analysis. Eur J Pediatr..

[13] Prummel MF, Strieder T, Wiersinga WM (2004). The environment and autoimmune thyroid diseases. Eur. J. Endocrinol..

[14] Costela-Ruiz VJ, Illescas-Montes R, Puerta-Puerta JM, Ruiz C, Melguizo-Rodríguez L (2020). SARS-CoV-2 infection: the role of cytokines in COVID-19 disease. Cytokine Growth Factor Rev..

[15] El Sabbagh R, Azar NS, Eid AA, Azar ST (2020). Thyroid dysfunctions due to immune checkpoint inhibitors: a review. Int J. Gen. Med..

[16] Bragazzi NL, Hejly A, Watad A, Adawi M, Amital H, Shoenfeld Y (2020). ASIAsyndrome and endocrine autoimmune disorders. Best Practice Research Clinical Endo-crinology Metabolism..

[17] Sharma A, Stan MN (2019). Thyrotoxicosis: Diagnosis and Management. Mayo Clinic Proceedings..

[18] Žarković M (2012). The role of oxidative stress on the pathogenesis of Graves‘disease. Journal of Thyroid Research. J Thyroid Res..

[19] Smith TJ (2010). Pathogenesis of Graves’ orbitopathy: a 2010 update. Journal of endo-crinological investigation..

